# Influence of ionic liquid composition on surface enrichment of fluorine-free Ru complexes

**DOI:** 10.1039/d5ra05316a

**Published:** 2025-09-30

**Authors:** Alisson Ceccatto, Luciano Sanchez Merlinsky, Luis M. Baraldo, Federico J. Williams, Florian Maier, Hans-Peter Steinrück

**Affiliations:** a Lehrstuhl für Physikalische Chemie 2, Friedrich-Alexander-Universität Erlangen-Nürnberg Egerlandstr. 3 91058 Erlangen Germany hans-peter.steinrueck@fau.de alisson.ac.ceccatto@fau.de; b Departamento de Química Inorgánica, Analítica y Química Física, Facultad de Ciencias Exactas y Naturales, Universidad de Buenos Aires Buenos Aires Argentina; c Instituto de Química Física de los Materiales, Medio Ambiente y Energía, CONICET-Universidad de Buenos Aires Buenos Aires Argentina

## Abstract

Tailoring the surface composition of ionic liquids (ILs) is a key strategy for enhancing the performance of supported ionic liquid phase (SILP) catalysts. Here, we investigate the interfacial behavior of fluorine-free Ru polypyridyl complexes functionalized with *n*-nonyl side chains (Ru–C_9_) dissolved in two ILs with contrasting physicochemical properties, such as hydrophobicity and surface tension, namely [C_4_C_1_Im][PF_6_] and [C_4_C_1_Im][OAc]. Using angle-resolved X-ray photoelectron spectroscopy (ARXPS), we show that Ru–C_9_ complexes undergo pronounced surface enrichment in [C_4_C_1_Im][PF_6_], adopting a buoy-like orientation at the IL/vacuum interface. Surface saturation is achieved at a bulk concentration of 0.12%_mol_, a regime relevant for efficient SILP catalysis. In contrast, no interfacial enrichment is observed in [C_4_C_1_Im][OAc], highlighting the critical role of IL composition, particularly anion identity and surface tension, in governing the surface distribution of metal complexes. These findings provide molecular-level insights into the design of IL-based catalytic systems with optimized interfacial properties.

## Abstract 2

Tailoring the interfacial behavior of dissolved fluorine-free Ru polypyridyl complexes functionalized with *n*-nonyl side chains by the choice of the solvent ionic liquid.

## Graphical abstract

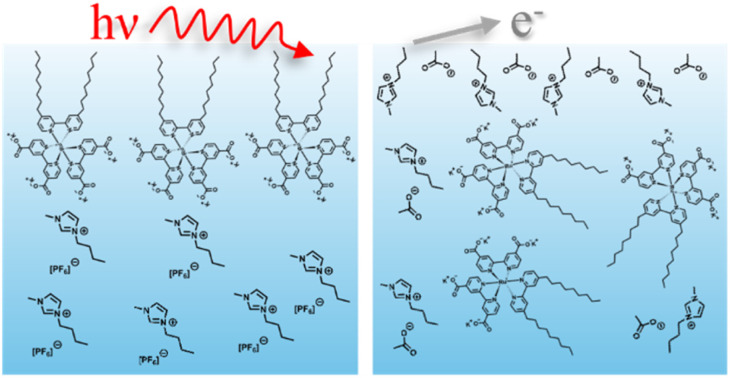

## Introduction

Ionic liquids (ILs) are salts with low melting points, typically below 100 °C, that exhibit key physicochemical properties desirable for catalysis, such as non-flammability, low vapour pressure, and a wide range of viscosities.^1–10^ This combination of properties makes ILs suitable materials for various applications, ranging from batteries^11,12^ to catalysis.^13,14^ In the field of catalysis, ILs have given rise to two notable concepts: supported ionic liquid phase (SILP)^15,16^ and solid catalyst with ionic liquid layer (SCILL).^17^ In the SILP concept, the catalyst consists of transition-metal complexes dissolved in a thin film of ionic liquid supported on a high-surface-area porous solid. Thus, SILP catalysts combine the advantages of homogeneous and heterogeneous catalysts.

In SILP catalysis, the ionic liquid/gas interface plays a crucial role in determining catalytic performance. Recent studies have demonstrated that the concentration of catalyst at this interface can be tuned through appropriate ligand design, enabling the catalyst complexes to be either homogeneously distributed throughout the solution or preferentially localized at the surface.^18–21^ The latter case reflects surface enrichment, which is expected to enhance the efficiency of catalytic processes. Consequently, understanding the key factors that govern the surface enrichment of catalysts in ILs is essential for optimizing their catalytic performance.

In this context, ionic liquids are ideal materials for investigating interfacial mechanisms due to their extremely low vapor pressure,^8^ which allows the use of surface science techniques under ultrahigh vacuum (UHV) conditions. Recently, angle-resolved X-ray photoelectron spectroscopy (ARXPS) has been employed to study the composition and orientation of ILs under UHV.^22,23^ Notably, increasing attention has been devoted to the surface enrichment of ILs containing dissolved metal complexes, see ref. 21 and references therein. For example, some of us recently reported that incorporating perfluorinated groups into Pt(II) complexes results in their preferential orientation toward the IL/vacuum interface, a phenomenon known as the “buoy effect”.^24^ A separate study has shown that Ru complexes free of per- and polyfluoroalkyl substances (PFAS), but functionalized with trioctylphosphine groups, exhibit similar surface enrichment behavior in [C_2_C_1_Im][Tf_2_N], with the hydrophobic phosphine ligands orienting toward the interface.^20^

Building on these insights, we reported the design of a series of Ru polypyridyl complexes functionalized with various alkyl chains at their periphery.^25^ By tuning the shape and length of these chains, we showed that complexes bearing *n*-nonyl substituents (Ru–C_9_) undergo pronounced surface enrichment in [C_2_C_1_Im][OAc]. Extending this investigation to ILs with different combinations of cations and anions is critical for developing a deeper molecular understanding of the factors governing interfacial behavior.

**Fig. 1:**
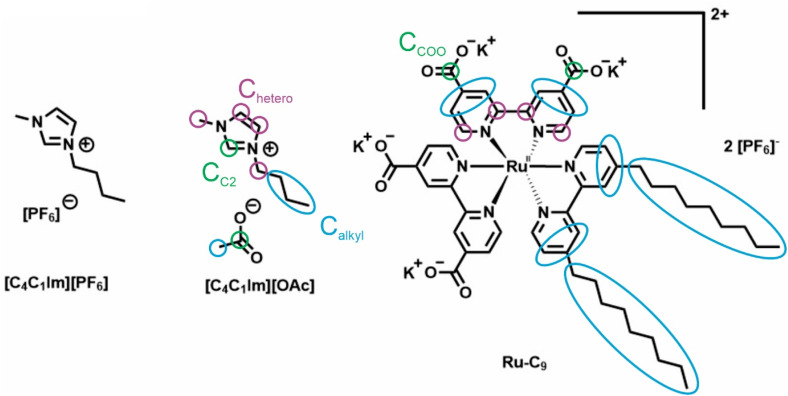
Molecular structures of the ILs and the complex investigated in this work, with the different C species assigned. The Ru complexes were synthesized as K^+^ and [PF_6_]^−^ salts.

In the present study, we explore the surface distribution of Ru–C_9_ complexes for two additional ILs, which have very different physicochemical properties, namely the hydrophobic [C_4_C_1_Im][PF_6_] and the hydrophilic [C_4_C_1_Im][OAc] ionic liquids (see Fig. 1).^26^ Since the surface tension of the pure solvent IL was found to influence surface enrichment of dissolved metal complexes,^21^ the two ILs were also chosen by their very different surface tension values (for values and references, see the last section). Angle-resolved X-ray photoelectron spectroscopy (ARXPS) measurements were carried out at both normal (0°) and grazing (80°) emission angles, enabling simultaneous analysis of bulk and surface composition under ultrahigh vacuum conditions. At 0°, the information depth is 7.0–9.0 nm, while at 80°, it is 1.0–1.5 nm, allowing for the investigation of the topmost layers at the IL/vacuum interface.^10,21^

Our results demonstrate a clear dependence of surface enrichment on IL composition. While Ru–C_9_ exhibits buoy-like behavior in [C_2_C_1_Im][OAc], no surface segregation is observed in [C_4_C_1_Im][OAc], highlighting the influence of the IL cation's alkyl chain length. Conversely, a marked surface enrichment re-emerges in [C_4_C_1_Im][PF_6_], underscoring the critical role of the IL anion in modulating interfacial organization. These findings provide valuable insights for the rational design of IL-based catalytic systems.

## Results and discussion

**Fig. 2:**
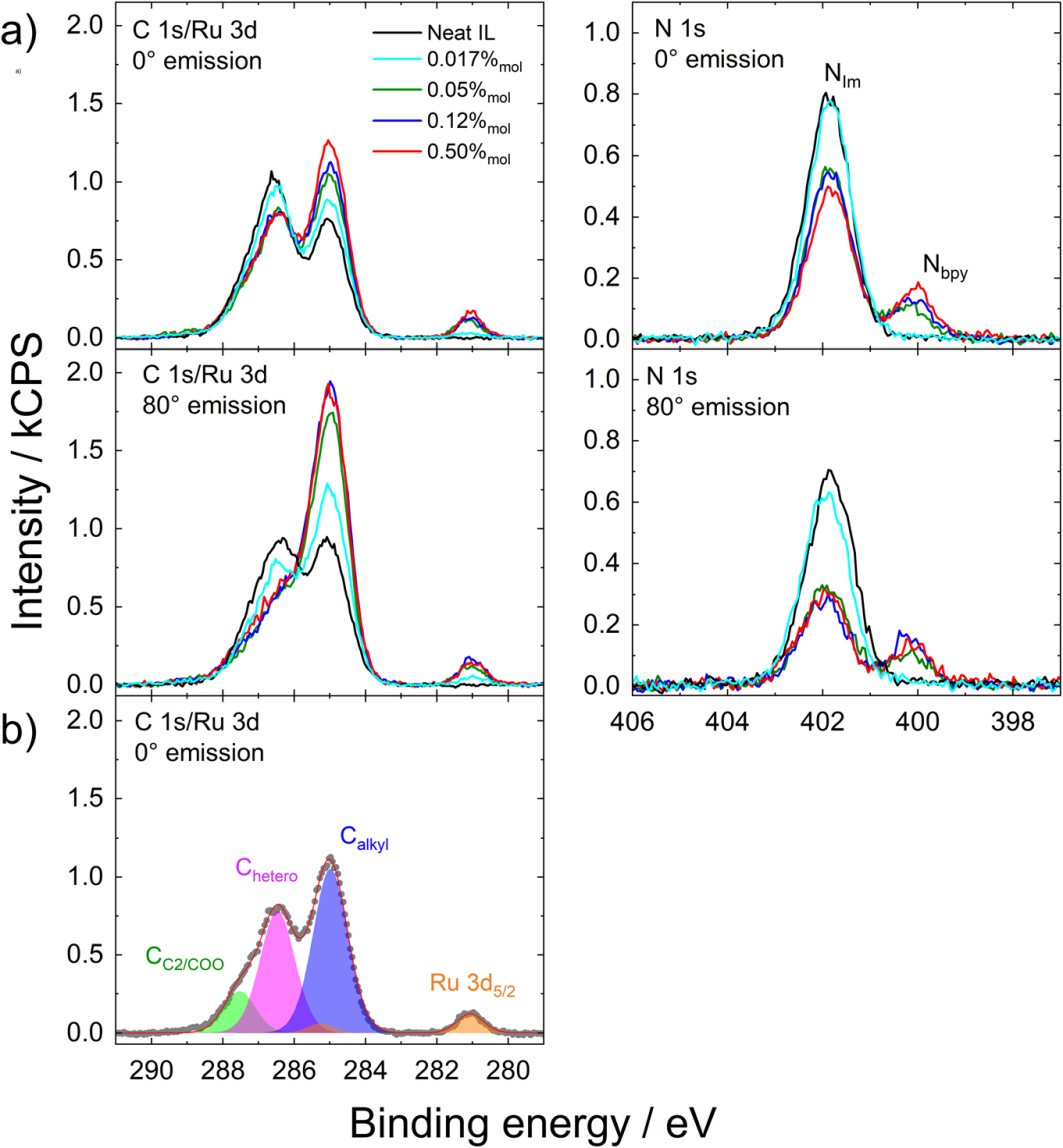
(a) XP spectra for C 1s/Ru 3d and N 1s regions of 0.017%_mol_ (cyan), 0.05%_mol_ (green), 0.12%_mol_ (blue), and 0.50%_mol_ (red) of Ru–C_9_ in [C_4_C_1_Im][PF_6_] at 0° (top row) and 80° (bottom row) emission. The neat [C_4_C_1_Im][PF_6_] is plotted in black for comparison. (b) Fitting of C 1s/Ru 3d peaks of 0.12%_mol_ solution of Ru–C_9_ in [C_4_C_1_Im][PF_6_] at 0° emission, with the assigned species considered in the fitting.

ARXPS measurements were performed on Ru–C_9_ solutions in [C_4_C_1_Im][PF_6_] with concentrations ranging from 0.50%_mol_ down to 0.017%_mol_. Fig. 2a shows the concentration-dependent C 1s/Ru 3d and N 1s XP spectra measured simultaneously at normal and grazing emission angles, along with the spectra of neat [C_4_C_1_Im][PF_6_] (black spectra) shown for reference. The complete XP spectra, including all relevant elemental regions, are provided in Fig. S1–S4 of the SI. The C 1s/Ru 3d spectrum shows two main features: a small peak at ∼281.1 eV attributed to Ru 3d_5/2_ and a broad peak centered around 286 eV, corresponding mainly to carbon species with a very small contribution of the Ru 3d_3/2_ spin–orbit doublet peak at ∼285.3 eV. Fig. 2b displays the detailed fitting for the 0.12%_mol_ solution at 0° emission. In line with previous work,^25^ the C 1s signal was fitted with three main components: the imidazolium C_2_ carbon and carboxylate groups at 287.5 eV (green), C atoms bonded to heteroatoms (C_hetero_) at 286.5 eV (purple), and aliphatic (C_alkyl_) carbons at 285.0 eV (blue). The Ru 3d_5/2_ peak appears at 281.1 eV, consistent with a +2 oxidation state of the Ru center.^27^ Two distinct nitrogen species are resolved in the N 1s spectra shown in Fig. 2a: the more intense peak at 401.9 eV corresponds to the imidazolium nitrogen atoms in the IL (N_Im_), while the less intense peak at 400.0 eV is assigned to the bipyridine nitrogen atoms (N_bpy_) coordinated to Ru. The presence of K^+^ counterions in solution is confirmed by the K 2p signal, shown in Fig. S1–S4. Note that the XP spectrum of the solid Ru–C_9_ complex in Fig. S5 also shows the presence of K^+^ counterions associated with the carboxylate groups.

A striking feature of the ARXPS data shown in Fig. 2 is the high intensity of the Ru 3d_5/2_ and N_bpy_ signals under bulk-sensitive conditions (0° emission angle), which show a further slight enhancement under surface-sensitive conditions (80° emission angle) (Table S1). The quantitative analysis shown in Table S1a–d in the SI for the 0.50%_mol_, 0.12%_mol_, 0.05%_mol_, and 0.017%_mol_ solutions reveals a strongly enhanced intensity of the Ru 3d_5/2_ and N_bpy_ signals relative to nominal values, suggesting that the complexes are located in the outermost layer at the IL/vacuum interface. A similar trend is observed for the C_alkyl_ signal, partially originating from the complex, which exhibits an even more pronounced increase at 80°, consistent with strong surface enrichment of the complex at the IL/vacuum interface. Notably, the greater enhancement of the C_9_-derived C_alkyl_ signal compared to Ru 3d_5/2_ and N_bpy_ suggests that within the outermost layer, the terminal alkyl chains of the complexes preferentially orient toward the interface, while the metallic core orients toward the bulk of the IL. This implies that the C_9_ chains act as surface-directing groups, anchoring the complex at the interface. Further supporting this interfacial enrichment, signals associated with the IL (*e.g.*, C_2_, C_hetero_ and N_Im_) are attenuated relative to expected values, indicating a depletion of [C_4_C_1_Im]^+^ from the surface. The most compelling evidence comes from the IL-specific N_Im_ signal in 0°, which for concentrations above 0.017%_mol_ is considerably decreased as compared to the nominal value; this effect is even more pronounced in 80°, confirming that the IL ions are strongly depleted from the outermost layer.

**Fig. 3:**
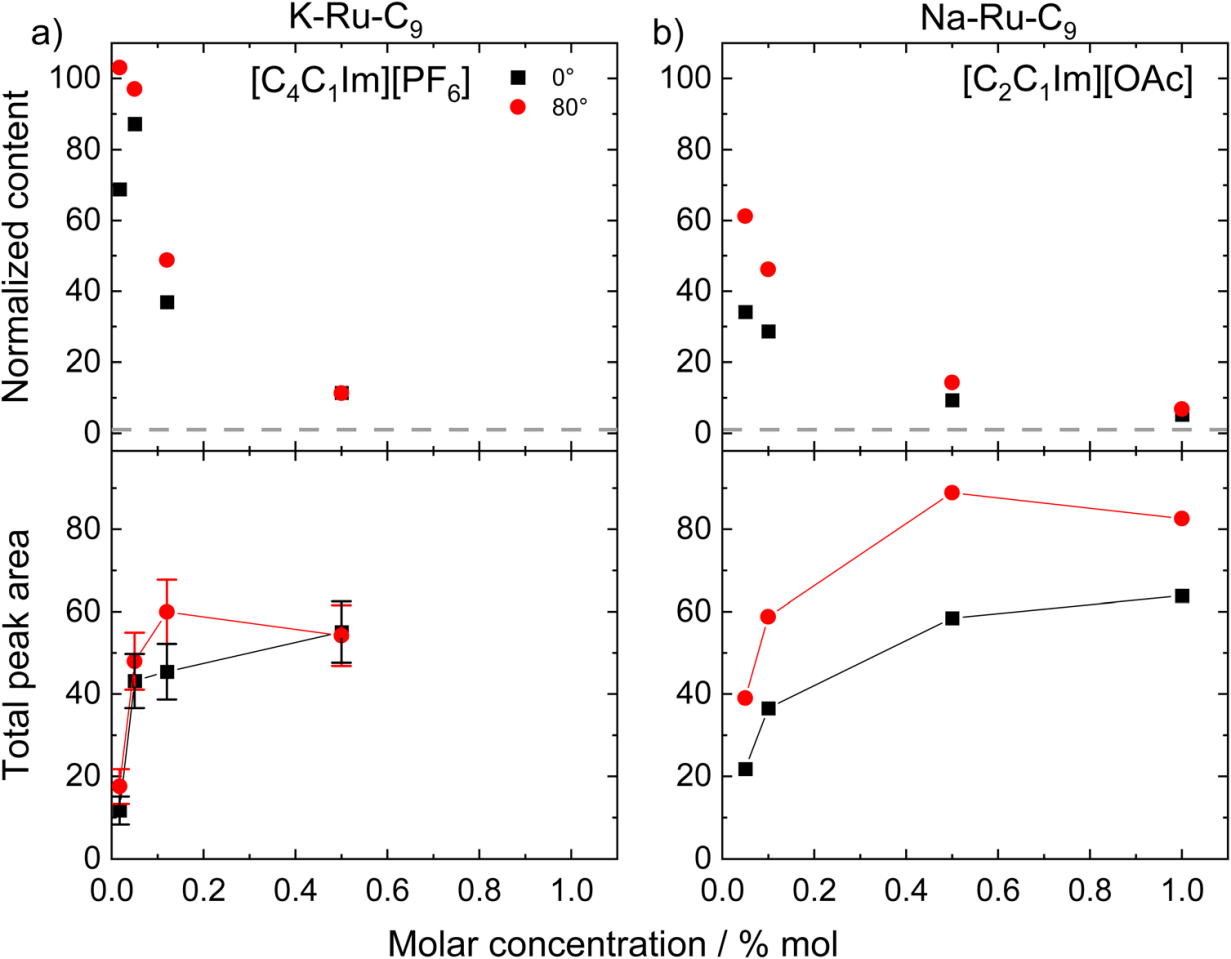
Normalized content obtained from Ru 3d_5/2_ signal at 0° and 80° (top) and the absolute intensity of the Ru 3d_5/2_ signal (bottom) for solutions of (a) K–Ru–C_9_ in [C_4_C_1_Im][PF_6_] with concentrations ranging from 0.017%_mol_ to 0.50%_mol_ and (b) Na–Ru–C_9_ in [C_2_C_1_Im][OAc] with concentrations ranging from 0.05%_mol_ to 0.50%_mol_ (adapted from ref. 25 with permission). The error bars given in (a) are a reasonable estimate considering uncertainties in signal intensity and background subtraction.

Fig. 3a presents the Ru content (see Table S1), normalized to the nominal value (top panel), and the Ru 3d_5/2_ peak area (bottom panel) for both emission angles (0° and 80°), as a function of the Ru–C_9_ concentration in [C_4_C_1_Im][PF_6_]. The dashed line at a normalized value of 1 corresponds to the case where Ru-complexes are homogeneously distributed and randomly oriented within the ionic liquid (IL). In both bulk-sensitive (0°) and surface-sensitive (80°) measurements, the normalized Ru contents increase as the complex concentration decreases, indicating a pronounced surface enrichment at lower concentrations. At 80°, the enrichment factor is ∼100 for the 0.017%_mol_ solution and decreases to ∼11 for the 0.50%_mol_ solution. From the absolute Ru 3d signal intensity, depicted in Fig. 3a (bottom panel), we conclude that the complex concentration at the surface reaches saturation already at ∼0.1%_mol_.

For comparison, Fig. 3b displays previously reported results^25^ for Ru–C_9_ in [C_2_C_1_Im][OAc]. In that earlier study, Na^+^ was used instead of K^+^ as the countercation in the Ru–C_9_ complex, which could be labelled Na–Ru–C_9_ or K–Ru–C_9_; as will be discussed later, this substitution has negligible influence on surface segregation. Overall, the behavior of the complex in the two different solvent ILs is quite similar. The most notable difference is that for Ru–C_9_ in [C_4_C_1_Im][PF_6_], saturation is reached at a lower complex concentration: in [C_4_C_1_Im][PF_6_], at 0.05%_mol_ already ∼80% of the saturation value is reached, while the corresponding value in [C_2_C_1_Im][OAc] is only ∼40%. Furthermore, the enhancement of the 80° signal as compared to the 0° signal is less pronounced for [C_4_C_1_Im][PF_6_], indicating a more pronounced orientation of the complex with the alkyl chains towards the vacuum side and the Ru center towards the bulk solvent IL (leading to an attenuation of the Ru signal at 80°).

**Fig. 4:**
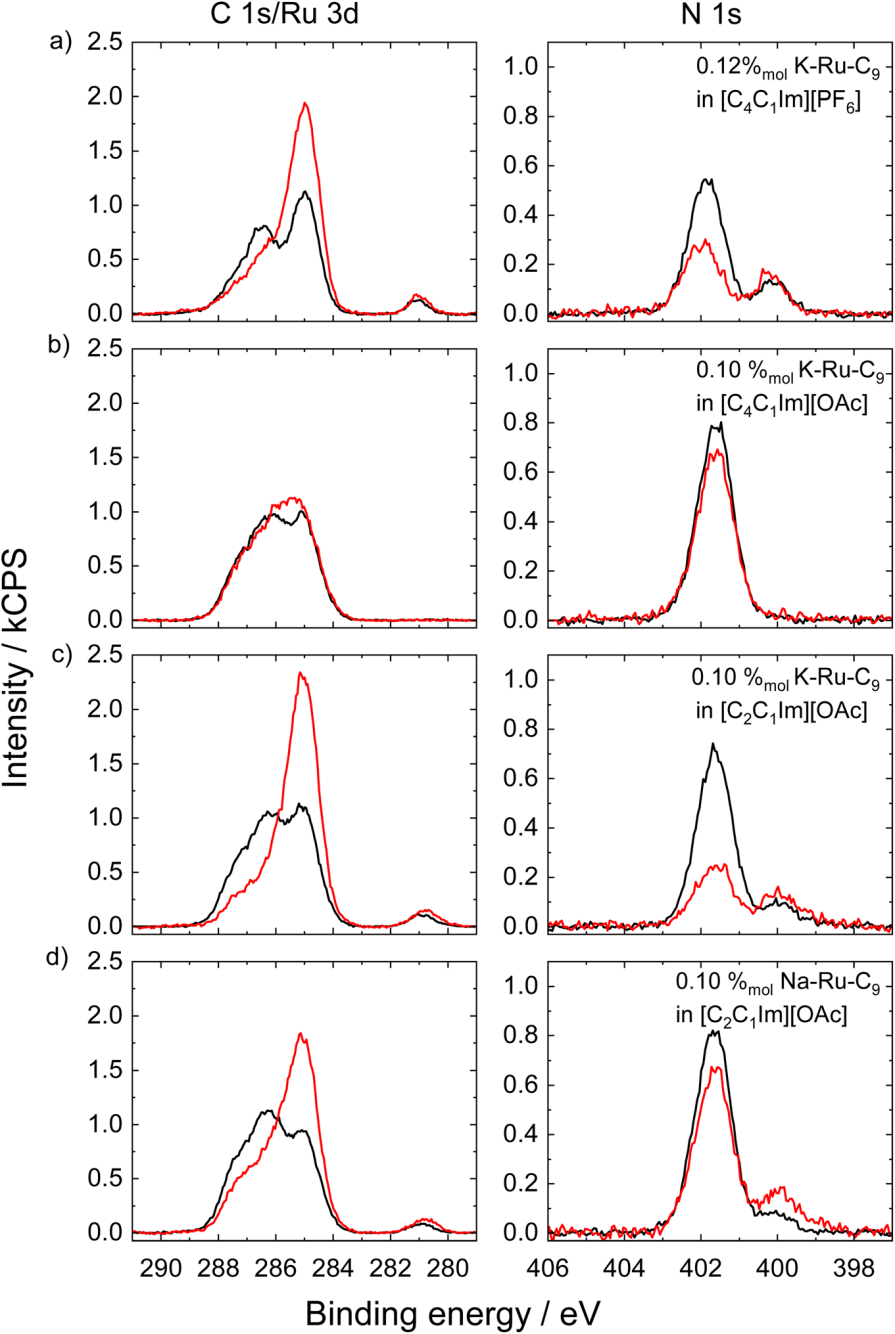
XP spectra at 0° (black) and 80° (red) for the C 1s/Ru 3d (left) and N 1s (right) regions for (a) 0.12%_mol_ solution of K–Ru–C_9_ complexes in [C_4_C_1_Im][PF_6_], 0.10%_mol_ solution of (b) K–Ru–C_9_ in [C_4_C_1_Im][OAc], (c) K–Ru–C_9_ in [C_2_C_1_Im][OAc], and (d) Na–Ru–C_9_ in [C_2_C_1_Im][OAc] (adapted from ref. 25, with permission).

Fig. 4 presents the C 1s/Ru 3d and N 1s XP spectra acquired simultaneously at 0° (black) and 80° (red) emission angles for the K–Ru–C_9_ complex dissolved in (a) [C_4_C_1_Im][PF_6_], (b) [C_4_C_1_Im][OAc], (c) [C_2_C_1_Im][OAc], and for the Na–Ru–C_9_ complex in (d) [C_2_C_1_Im][OAc], with concentration of 0.12 or 0.10%_mol_. As previously discussed, the K–Ru–C_9_ complex in [C_4_C_1_Im][PF_6_] exhibits pronounced surface enrichment, characterized by a surface layer terminated by C_9_ chains and a depletion of IL cations at the IL/vacuum interface. This is evidenced by the marked increase in the C_alkyl_ signal and the strong attenuation of the N_Im_ signal at 80° (Fig. 4a). In contrast, the behavior of K–Ru–C_9_ in [C_4_C_1_Im][OAc] in Fig. 4b is markedly different: no Ru 3d_5/2_ or N_bpy_ signals are detected (not even for a concentration of 0.50%_mol_; for the full sets of spectra of 0.10%_mol_ and 0.50%_mol_ K–Ru–C_9_ in [C_4_C_1_Im][OAc] see Fig. S7 and S8, respectively, in the SI), indicating that the complex is absent (not detectable) from the IL/vacuum interface (see quantitative analysis in Table S1e and f, in the SI). Additionally, a modest increase in the C_alkyl_ signal at 80° suggests that the [C_4_C_1_Im]^+^ cations orient at the interface with their butyl chains preferentially pointing toward the vacuum, as evident from the C 1s signal for the neat [C_4_C_1_Im][OAc] IL in Fig. S9 in the SI (for comparison, see also quantitative analysis in Table S1g, in the SI). Interestingly, shortening the alkyl chain length alters the interfacial behavior of the complex. In [C_2_C_1_Im][OAc], both K–Ru–C_9_ and Na–Ru–C_9_ show enhanced XPS signals for C_alkyl_, N_bpy_, and Ru 3d_5/2_ at 80°, as seen in Fig. 4c and d, alongside a decrease in the IL-related signals (C_hetero_ and N_Im_). This now again indicates surface enrichment of the Ru–C_9_ complex, with the nonyl chains preferentially orienting toward the interface. Finally, note that the intensity differences observed between Fig. 4c and d – particularly of the N_Im_ signal at 401.9 eV – can be attributed to the significant presence of K^+^ counterions at the surface as measured by ARXPS (see Fig. S6 of SI), which dampens the N_Im_ signal, while at similar bulk concentrations Na^+^ counterions near the surface have a concentration below the detection limit of XPS (see Table S2 in SI to ref. 25); this implies the presence of more [C_2_C_1_Im]^+^ near the surface in case of the Na–Ru–C_9_ complex and thus, less damping of the N_Im_ signal, in particular at 80°. Importantly, these counterion-specific effects do not alter the overall surface enrichment of the Ru complexes, as comparable Ru 3d_5_/_2_ and N_bpy_ signals are observed for both K^+^ and Na^+^.

To summarize, the Ru–C_9_ complexes exhibit a buoy-like behavior in [C_4_C_1_Im][PF_6_] and [C_2_C_1_Im][OAc], whereas no surface enrichment is observed in [C_4_C_1_Im][OAc]. This difference can be attributed to variations in the surface tension (ST) of the ionic liquids: [C_4_C_1_Im][OAc] has a surface tension of 37.5 mN m^−1^ at 296 K,^28^ compared to 43.4 mN m^−1^ for [C_4_C_1_Im][PF_6_] at 298 K (ref. 29) and 46.0 mN m^−1^ for [C_2_C_1_Im][OAc] at 294 K.^23^ The surface tension of ILs depends on the chemical nature of the ion pairs. Increasing the alkyl chain length of either cations or anions generally lowers the surface tension, as observed here for the [OAc]-based ILs. In addition, the surface affinity of the anions plays a key role, as is evident from the lower surface tension of [C_4_C_1_Im][OAc] as compared to [C_4_C_1_Im][PF_6_]. Typically, anions of ILs with lower surface tension are more surface-active, as deduced from ARXPS studies on IL mixtures with different anions but the same cation.^23^ In other words, the [PF_6_]^−^ anions are less surface-active than the [OAc]^−^ anions. This difference enhances the surface cohesive energy of the [PF_6_]–IL, resulting in higher surface tension for ILs containing [PF_6_]^−^ compared to [OAc]^−^, which results in a higher driving force for surface enrichment of the complex. This interpretation aligns with previous observations of Pt complexes in [PF_6_]-based ILs, where surface enrichment was more pronounced in ILs with higher surface tension.^30^ Finally, we note that, as expected, replacing K^+^ with Na^+^ (compare Fig. 4c and d) has no noticeable effect on surface segregation.

## Conclusions

Angle-resolved X-ray photoelectron spectroscopy measurements reveal that Ru–C_9_ polypyridyl complexes exhibit strong surface enrichment when dissolved in [C_4_C_1_Im][PF_6_] and [C_2_C_1_Im][OAc], driven by the segregation of their long hydrophobic nonyl chains toward the IL/vacuum interface. This buoy-like orientation results in a surface layer enriched in aliphatic carbon, while the metallic core of the complex remains closer to the bulk of the solvent IL. Notably, the extent of surface segregation is concentration-dependent: as the bulk concentration of Ru–C_9_ decreases, the enrichment factor increases significantly, reaching values of ∼100 at 0.017%_mol_, with surface saturation occurring already at 0.12%_mol_. These findings are especially relevant for SILP catalysis, where optimal catalyst utilization at low concentrations is desired.

In contrast, when the solvent is switched to [C_4_C_1_Im][OAc], no evidence of surface enrichment is observed, and the Ru complex is effectively excluded from the IL/vacuum interface. This striking difference in behavior is attributed to the surface tension of the ILs, consistent with previous studies where surface enrichment of metal complexes was favored in ILs with higher surface tension. Additionally, replacement of K^+^ with Na^+^ in the Ru–C_9_ complex does not affect surface behavior, confirming that the counterion has a negligible influence on interfacial segregation under these conditions.

Altogether, these results highlight the critical role of IL composition—particularly the structure of the cation and the surface activity of the anion—in governing the interfacial organization of molecular catalysts. Such insights are essential for the rational design of IL-based catalytic systems where maximizing interfacial activity at low loading is key.

## Experimental

### Materials and sample preparation

The Ru–C_9_ was prepared as described previously^25^ using KOH instead of NaOH.

1-Butyl-3-methylimidazolium hexafluorophosphate ([C_4_C_1_Im][PF_6_]), 1-butyl-3-methylimidazolium acetate ([C_4_C_1_Im][OAc]), 1-ethyl-3-methylimidazolium acetate ([C_2_C_1_Im][OAc]), and all other chemicals used in this work were commercially obtained and used without further purification.

The Ru–C_9_ solutions were prepared by stirring for 24 h under ambient conditions in the ILs. After the preparation, the solutions were introduced into the load lock system under UHV conditions and degassed for at least 12 h before measuring.

### ARXPS measurements and data evaluation

The relevant information about the processing and data evaluation of the XPS measurements can be found in the previous reports.^24^ For the ARXPS measurements, the dual analyzer system for surface analysis (DASSA) was used.^31^ The experimental setup has a unique geometry with two analyzers mounted at 0° and 80° with respect to the surface plane. The system allows the simultaneous measurements at different information depths (IDs), probing the material's “bulk” and “surface”. Similarly to previous work,^18^ the binding energy scale for all XP spectra was referenced to the C 1s signal of carbon atoms bonded to other C atoms (C_alkyl_) at 285.0 eV. The XP spectra at 80° were scaled up by the geometric factor to compensate for the lower intensity compared to 0°. This allows us to correlate any intensity difference as enrichment or depletion effects. Further information about this procedure can be found in the literature.^31^ We performed the quantitative analysis of the intensities using the atomic sensitivity factors (ASFs).^32^ The intensity detected in the XP spectra was normalized to the overall intensity (sum over all intensities corrected by the atomic sensitivity factors) of the neat [C_4_C_1_Im][PF_6_] IL at 0°.

## Author contributions

Conceptualization (HPS, FM, FJW, LMB), data curation (AC), formal analysis (AC), funding acquisition (HPS, FJW), investigation (AC, LSM), methodology (AC, FJW, FM, LSM, LMB, HPS), project administration (FM, HPS), resources (HPS), software (AC), supervision (HPS, FJW, LMB), validation (FJW, HPS, FM), visualization (AC), writing – original draft (AC), writing – review & editing (FJW, FM, HPS).

## Conflicts of interest

The authors declare that they have no conflict of interest.

## Supplementary Material

RA-015-D5RA05316A-s001

## Data Availability

Data for this article, including (XPS raw data and analysis) are available at Zenodo at https://doi.org/10.5281/zenodo.16306470. Supplementary information is available. See DOI: https://doi.org/10.1039/d5ra05316a.
