# Supplementary material for: Influence of ionic liquid composition on surface enrichment of fluorine-free Ru complexes
Source: RSC Adv. 2025 Sep 30;15(43):36137–44. doi: 10.1039/d5ra05316a (PMC12481246; doi:10.1039/d5ra05316a)
Supplement: RA-015-D5RA05316A-s001 [file RA-015-D5RA05316A-s001.pdf]

Supplementary Information (SI) for RSC Advances.
This journal is © The Royal Society of Chemistry 2025

Supporting Information

Influence of Ionic Liquid Composition on Surface Enrichment of Fluorine-Free Ru Complexes

Alisson Ceccatto[a],*, Luciano Sanchez Merlinsky[b,c], Luis M. Baraldo[b,c], Federico J. Williams[b,c], Florian Maier [a], Hans-Peter Steinrück [a]*

[a] Lehrstuhl für Physikalische Chemie 2, Friedrich-Alexander-Universität Erlangen-Nürnberg, Egerlandstr. 3, 91058 Erlangen, Germany

[b] Departamento de Química Inorgánica, Analítica y Química Física, Facultad de Ciencias Exactas y Naturales, Universidad de Buenos Aires, Buenos Aires, Argentina

[c] Instituto de Química Física de los Materiales, Medio Ambiente y Energía, CONICET-Universidad de Buenos Aires, Buenos Aires, Argentina

*Corresponding Authors: hans-peter.steinrueck@fau.de. alisson.ac.ceccatto@fau.de

CONTENTS:

Figure S1 – S9: XP spectra of all studied solutions

Table S1: Quantitative analysis of all studied solutions

0.017 $\%_{mol}$ of Ru-C_9 in [C_4C_1Im][PF_6]

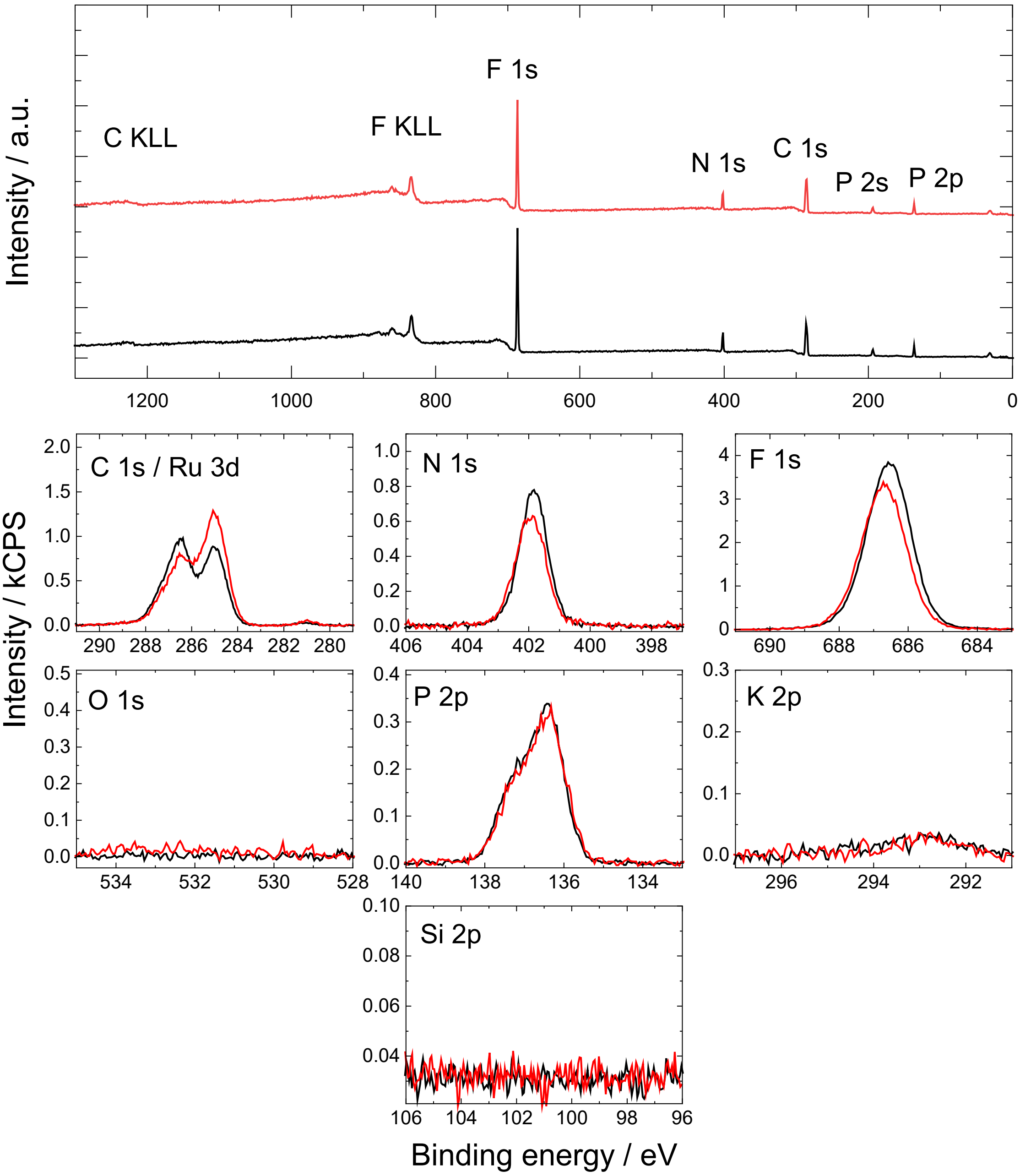

Figure S1: Survey, C 1s/Ru 3d, N 1s, F 1s, O 1s, P 2p, K 2p, and Si 2p XP spectra for 0.017 $\%_{mol}$ solution of Ru-C_9 in [C_4C_1Im][PF_6] at 0° (black) and 80° (red) emission.

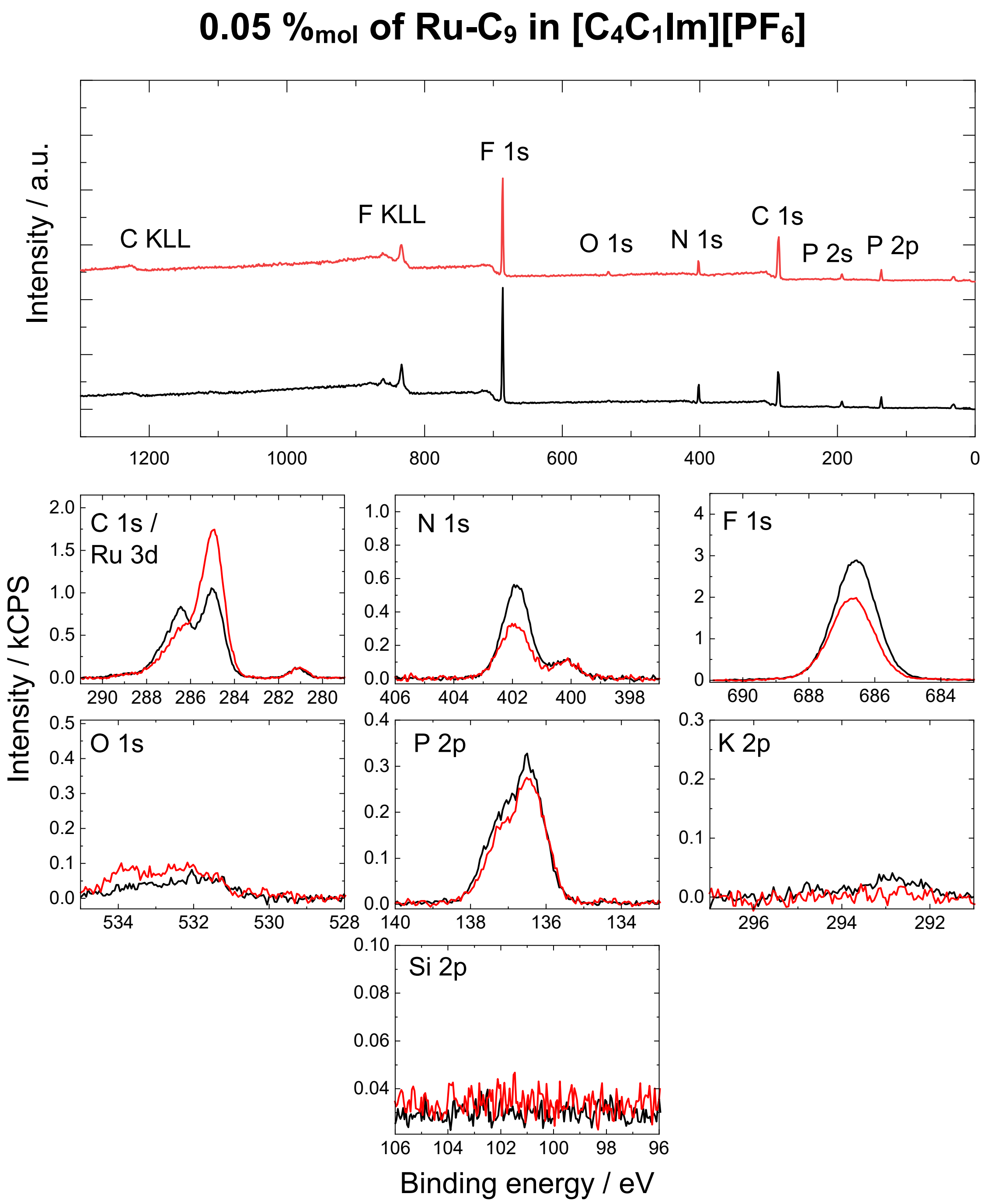

Figure S2: Survey, C 1s/Ru 3d, N 1s, F 1s, O 1s, P 2p, K 2p, and Si 2p XP spectra for 0.05 $\%_{mol}$ solution of Ru-C_9 in $[C_4C_1Im][PF_6]$, at 0° (black) and 80° (red) emission.

0.12 $\%_{mol}$ of Ru-C_9 in $[C_4C_1Im][PF_6]$

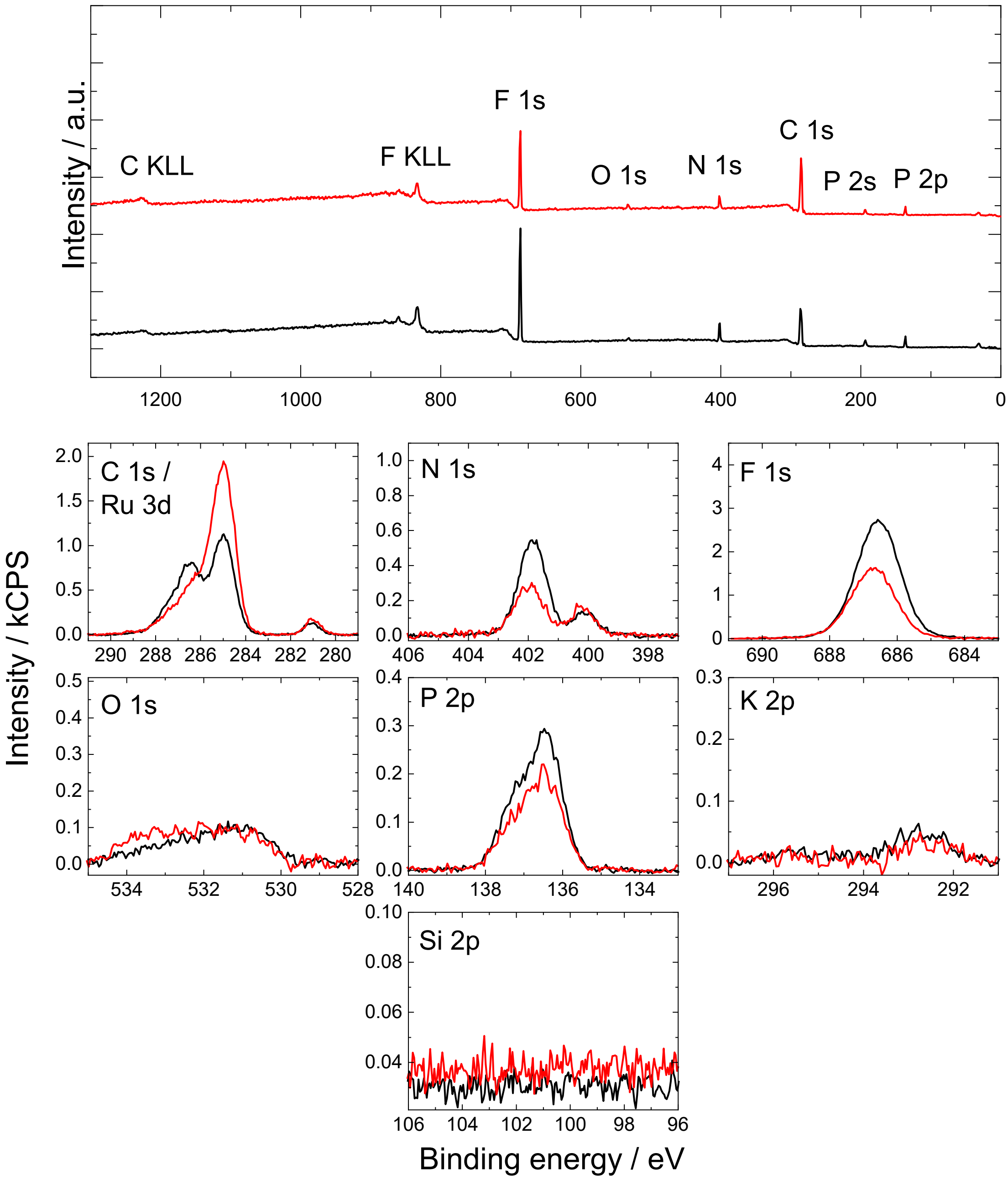

Figure S3: Survey, C 1s/Ru 3d, N 1s, F 1s, O 1s, P 2p, K 2p, and Si 2p XP spectra for 0.12 $\%_{mol}$ solution of Ru-C_9 in $[C_4C_1Im][PF_6]$ at 0° (black) and 80° (red) emission.

0.50 %$_{mol}$ of Ru-C_9 in [C_4C_1Im][PF_6]

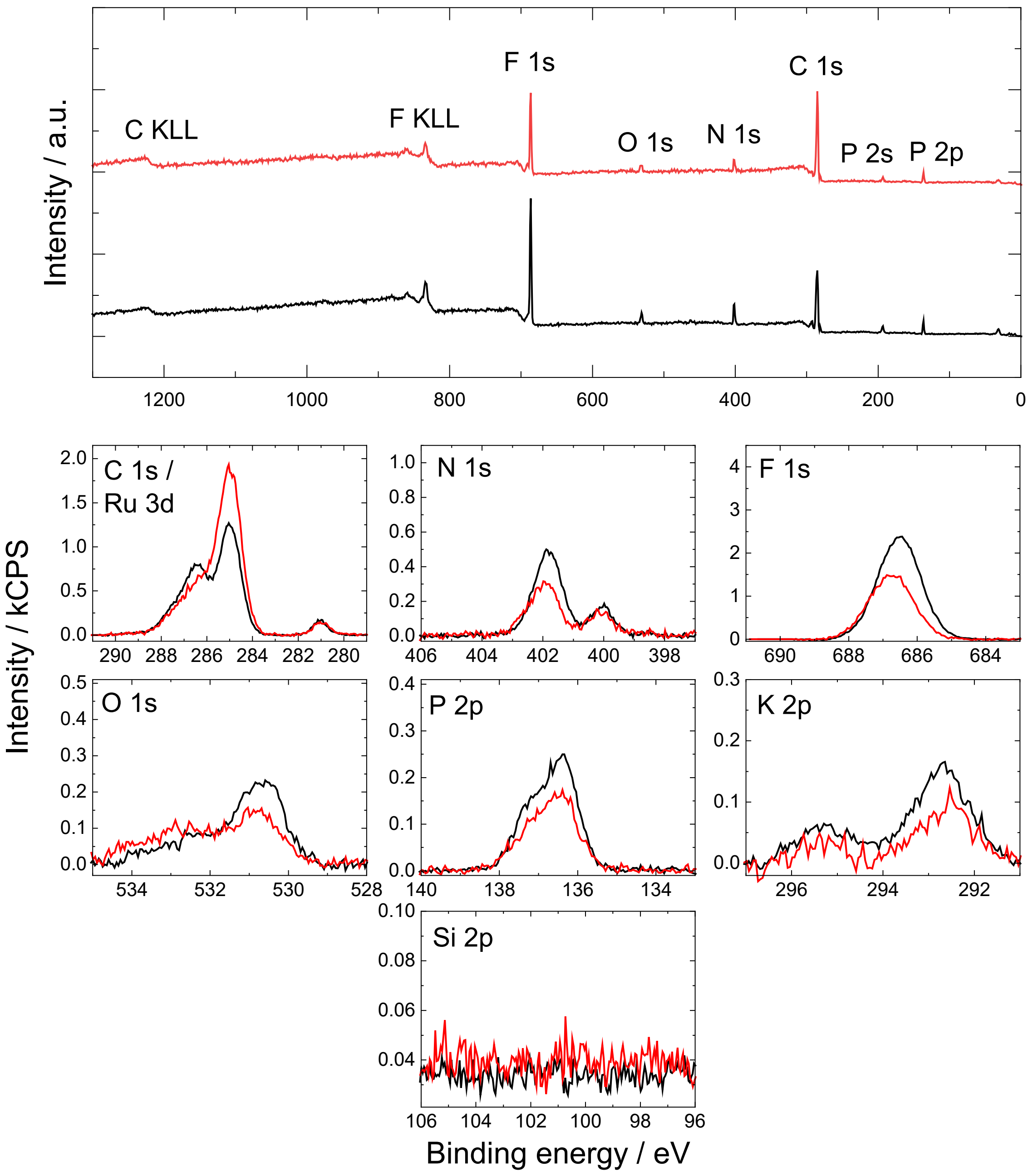

Figure S4: Survey, C 1s/Ru 3d, N 1s, F 1s, O 1s, P 2p, K 2p, and Si 2p XP spectra for 0.50 %$_{mol}$ solution of Ru-C_9 in [C_4C_1Im][PF_6] at 0° (black) and 80° (red) emission.

Neat Ru-C_9

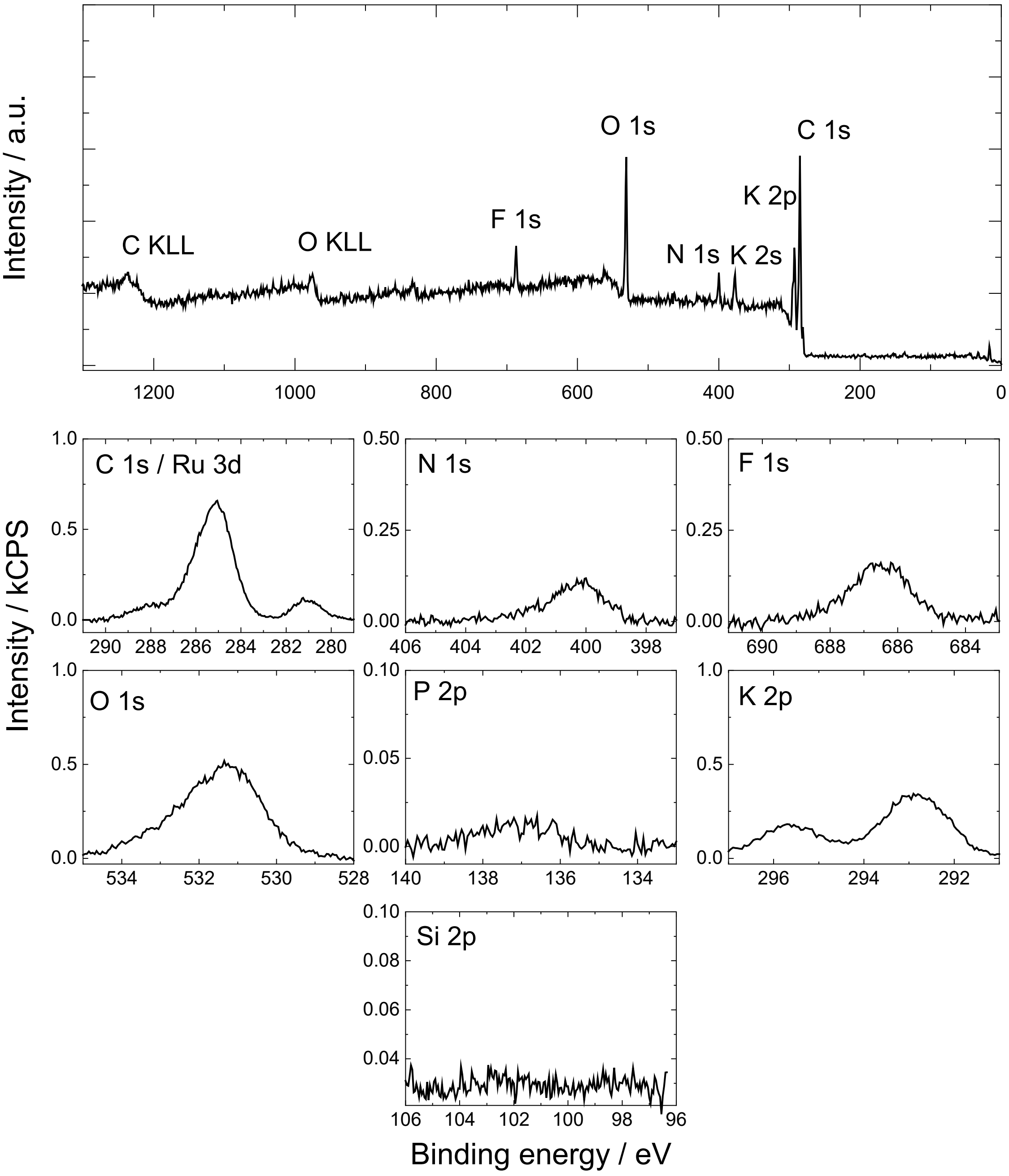

Figure S5: Survey, C 1s/Ru 3d, N 1s, F 1s, O 1s, P 2p, K 2p, and Si 2p XP spectra for the neat Ru-C_9 complexes at 0° emission.

0.10 %$_{mol}$ of Ru-C$_9$ in [C$_2$C$_1$Im][OAc]

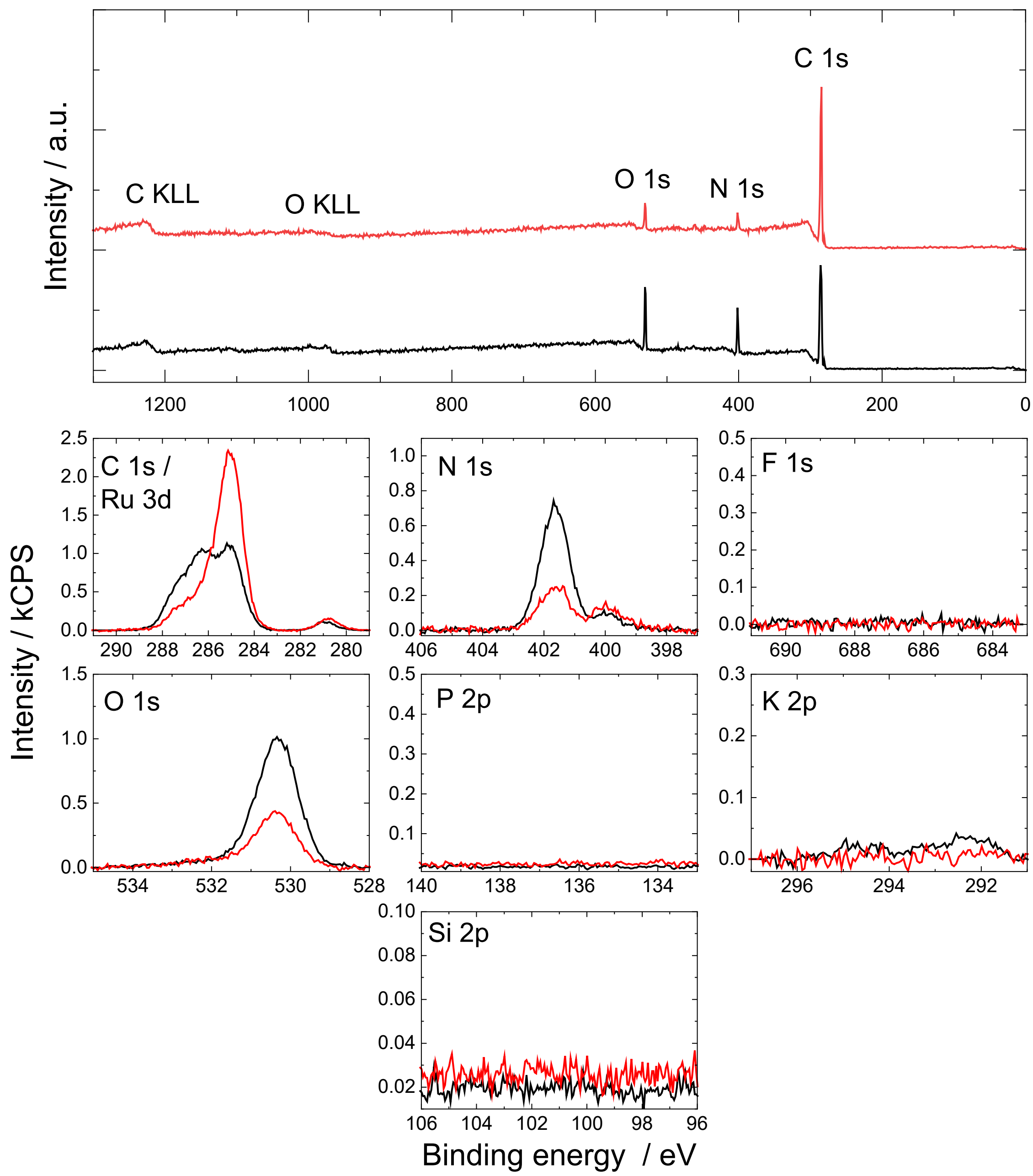

Figure S6: Survey, C 1s/Ru 3d, N 1s, F 1s, O 1s, P 2p, K 2p, and Si 2p XP spectra for 0.10 %$_{mol}$ solution of Ru-C$_9$ in [C$_2$C$_1$Im][OAc] at 0° (black) and 80° (red) emission.

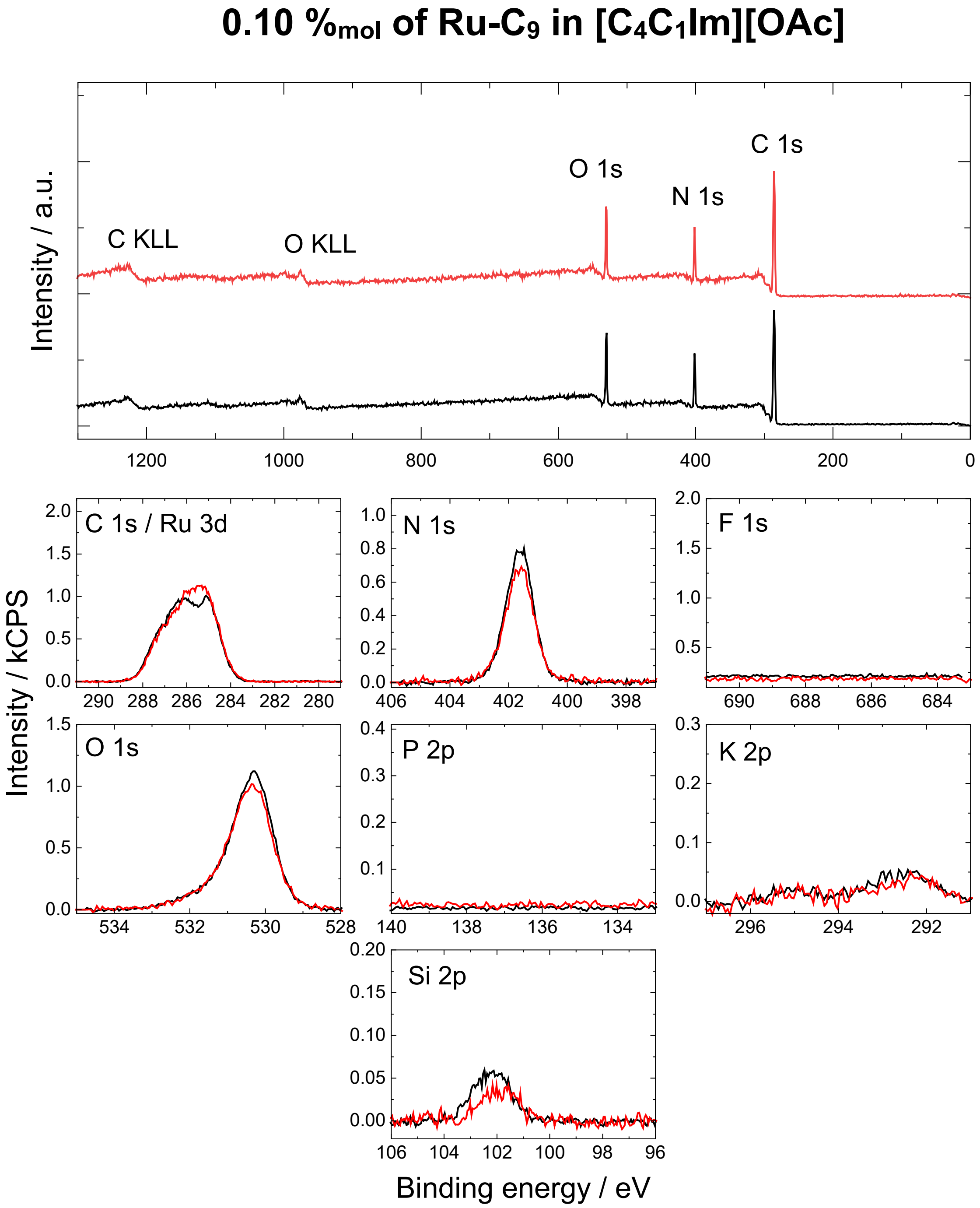

Figure S7: Survey, C 1s/Ru 3d, N 1s, F 1s, O 1s, P 2p, K 2p, and Si 2p XP spectra for 0.10 $\%_{mol}$ solution of Ru-C_9 in [C_4C_1Im][OAc] at 0° (black) and 80° (red) emission; note that in addition to the expected solution signals, a minor Si-containing contamination of unknown origin is also seen in the Si 2p region.

0.50 $\%_{mol}$ of Ru-C_9 in [C_4C_1Im][OAc]

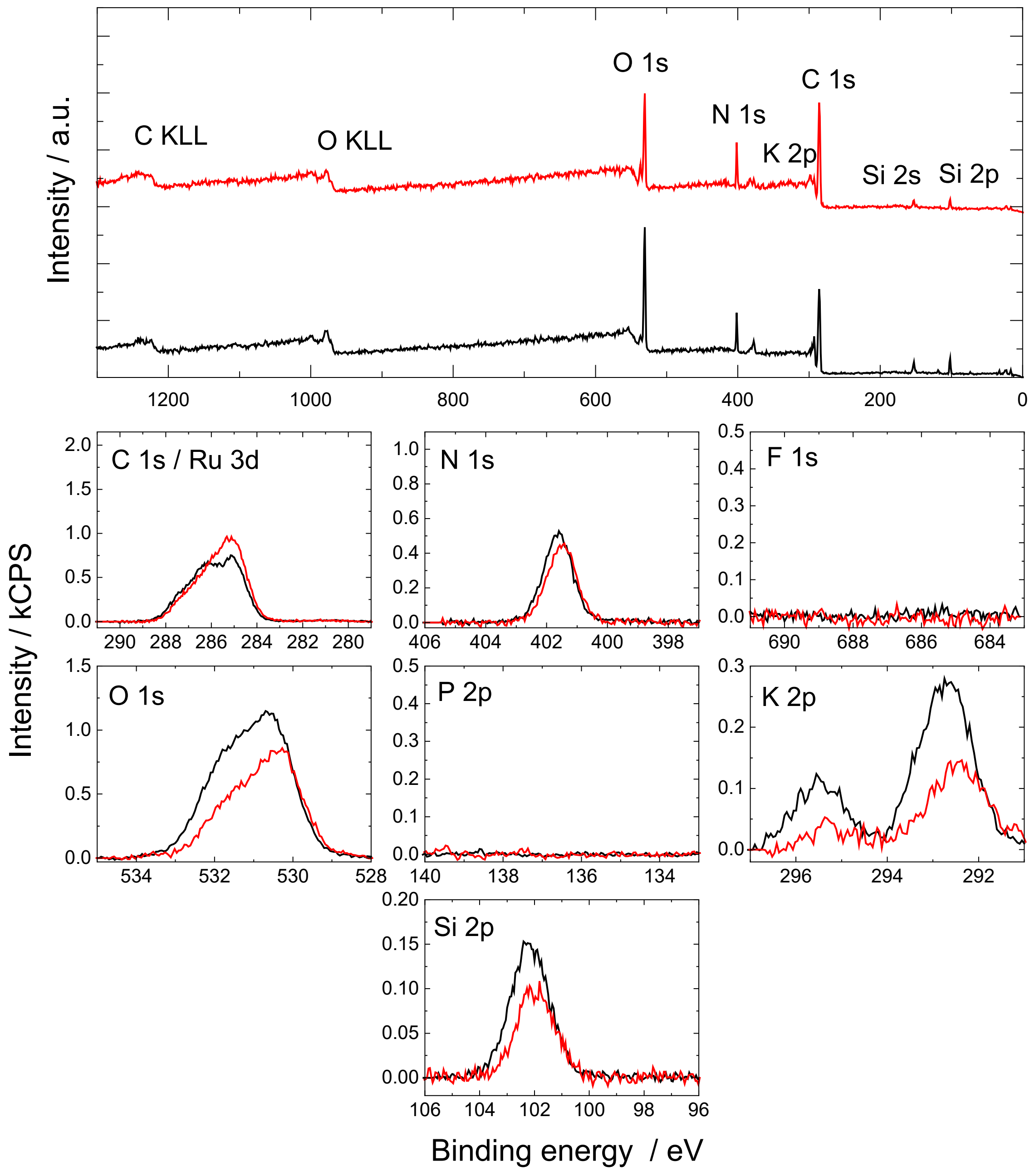

Figure S8: Survey, C 1s/Ru 3d, N 1s, F 1s, O 1s, P 2p, K 2p, and Si 2p XP spectra for 0.50 $\%_{mol}$ solution of Ru-C_9 in [C_4C_1Im][OAc] at 0° (black) and 80° (red) emission; note that in addition to the expected solution signals, a minor Si-containing contamination of unknown origin is also seen in the Si 2p region.

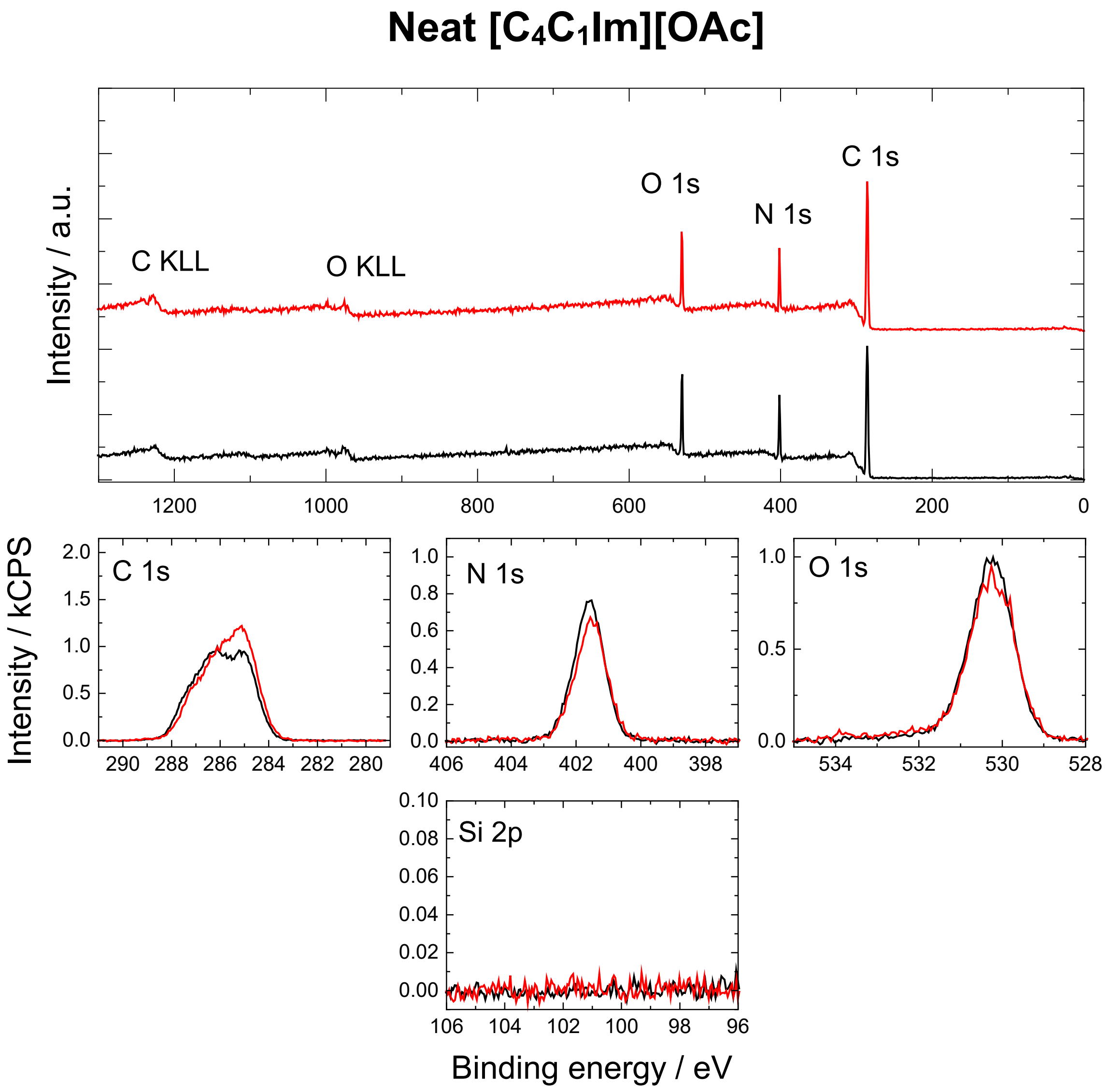

Figure S9: Survey, C 1s/Ru 3d, N 1s, O 1s, and Si 2p XP spectra for the neat [C_4C_1Im][OAc] IL at 0° (black) and 80° (red) emission.

Table S1: Quantitative analysis of the XPS core levels for the solutions of Ru-C_9 in $[C_4C_1Im][PF_6]$ with concentrations of a) 0.017 $\%_{mol}$, b) 0.05 $\%_{mol}$, c) 0.1 $\%_{mol}$ and d) 0.5 $\%_{mol}$, and in $[C_4C_1Im][OAc]$ with concentrations of e) 0.10 $\%_{mol}$ and f) 0.50 $\%_{mol}$; also included are the data for g) neat $[C_4C_1Im][PF_6]$. For species with signals below the detection limit, we assigned the symbol “<<”.

**a) 0.017 $\%_{mol}$ in $[C_4C_1Im][PF_6]$**	Ru $3d_{5/2}$	C 1s C_2	C 1s C_{het}	C 1s C_{alk}	N 1s N_{im}	N 1s N_{bpy}	O 1s O_{ac}	F 1s	P $2p_{3/2}$	K $2p_{3/2}$
BE/eV	281.1	287.5	286.5	285.0	401.9	399.9		686.6	136.4	292.9
Nominal	0.0002	1.00	4.00	3.01	2.00	0.001	0.001	6.00	1.00	0.0007
Exp. 0°	0.012	0.94	3.76	3.54	1.94	0.03		6.05	0.70	0.05
Exp. 80°	0.018	0.83	3.09	5.29	1.71	0.04		5.30	0.68	0.05
**b) 0.05 $\%_{mol}$ in $[C_4C_1Im][PF_6]$**										
BE/eV	281.1	287.5	286.5	285.0	401.9	400.2		686.6	136.4	292.8
Nominal	0.0005	1.00	4.01	3.02	2.00	0.003	0.004	6.01	1.00	0.002
Exp. 0°	0.043	0.95	3.54	4.66	1.53	0.29		5.10	0.72	0.05
Exp. 80°	0.048	0.83	2.59	8.04	0.98	0.29		3.59	0.64	
**c) 0.12 $\%_{mol}$ in $[C_4C_1Im][PF_6]$**										
BE/eV	281.1	287.5	286.5	285.0	401.9	400.1	530.9	686.6	136.4	292.8
Nominal	0.001	1.00	4.01	3.04	2.00	0.007	0.009	6.01	1.00	0.005
Exp. 0°	0.05	1.1	3.50	4.90	1.50	0.3	0.3	4.70	0.60	0.08
Exp. 80°	0.06	0.9	2.60	8.80	0.80	0.4	0.2	2.90	0.50	0.05
**d) 0.50 $\%_{mol}$ in $[C_4C_1Im][PF_6]$**										
BE/eV	281.1	287.5	286.5	285.0	401.8	400.1	530.7	686.5	136.4	292.7
Nominal	0.005	1	4.1	3.2	2	0.03	0.04	6.1	1	0.02
Exp. 0°	0.056	1.1	3.5	5.6	1.3	0.4	0.6	4.1	0.6	0.2
Exp. 80°	0.055	1.1	2.7	8.8	0.9	0.4	0.4	2.6	0.4	0.1
**e) 0.10 $\%_{mol}$ in $[C_4C_1Im][OAc]$**										
BE/eV		287.3	286.2	285.0	401.6		530.3			292.5
Nominal	0.001	2.0	4.0	4.0	2.0	0.006	2.01	0.012	0.002	0.004
Exp. 0°	<<	2.1	3.7	4.0	2.0	<<	2.13	<<	<<	0.08
Exp. 80°	<<	2.4	3.8	4.0	1.8	<<	2.02	<<	<<	0.06
**f) 0.50 $\%_{mol}$ in $[C_4C_1Im][OAc]$**										
BE/eV		287.4	286.2	285.0	401.6		530.3			292.7
Nominal	0.005	2.0	4.1	4.2	2.0	0.03	2.04	0.06	0.01	0.02
Exp. 0°	<<	1.7	3.6	4.1	1.8	<<	2.69	<<	<<	0.53
Exp. 80°	<<	1.6	3.2	5.5	1.6	<<	2.19	<<	<<	0.27
**g) Neat $[C_4C_1Im][OAc]$**										
BE/eV		287.3	286.3	285.0	401.6		530.3			
Nominal		2.0	4.0	4.0	2.0		2.00			
Exp. 0°		2.0	4.0	4.1	2.0		2.00			
Exp. 80°		1.9	3.7	4.7	1.8		1.90			
